# Flock House Virus RNA Polymerase Initiates RNA Synthesis De Novo and Possesses a Terminal Nucleotidyl Transferase Activity

**DOI:** 10.1371/journal.pone.0086876

**Published:** 2014-01-23

**Authors:** Wenzhe Wu, Zhaowei Wang, Hongjie Xia, Yongxiang Liu, Yang Qiu, Yujie Liu, Yuanyang Hu, Xi Zhou

**Affiliations:** State Key Laboratory of Virology, College of Life Sciences, Wuhan University, Wuhan, Hubei, China; University of California, Riverside, United States of America

## Abstract

Flock House virus (FHV) is a positive-stranded RNA virus with a bipartite genome of RNAs, RNA1 and RNA2, and belongs to the family *Nodaviridae*. As the most extensively studied nodavirus, FHV has become a well-recognized model for studying various aspects of RNA virology, particularly viral RNA replication and antiviral innate immunity. FHV RNA1 encodes protein A, which is an RNA-dependent RNA polymerase (RdRP) and functions as the sole viral replicase protein responsible for RNA replication. Although the RNA replication of FHV has been studied in considerable detail, the mechanism employed by FHV protein A to initiate RNA synthesis has not been determined. In this study, we characterized the RdRP activity of FHV protein A in detail and revealed that it can initiate RNA synthesis via a *de novo* (primer-independent) mechanism. Moreover, we found that FHV protein A also possesses a terminal nucleotidyl transferase (TNTase) activity, which was able to restore the nucleotide loss at the 3′-end initiation site of RNA template to rescue RNA synthesis initiation *in vitro*, and may function as a rescue and protection mechanism to protect the 3′ initiation site, and ensure the efficiency and accuracy of viral RNA synthesis. Altogether, our study establishes the *de novo* initiation mechanism of RdRP and the terminal rescue mechanism of TNTase for FHV protein A, and represents an important advance toward understanding FHV RNA replication.

## Introduction

Flock House virus (FHV) is a nonenveloped positive-strand (+) RNA viruses with a *T* = 3 icosahedral capsid, and belongs to genus Alphanodavirus of the family *Nodaviridae*. FHV was originally isolated from grass grubs in the former Flock House agricultural station in Bulls, Rangitikei District, New Zealand. Like other nodaviruses, FHV contains a bipartite genome of (+)-RNAs, RNA1 (3.1 kb) and RNA2 (1.4 kb), both of which are capped but not polyadenylated [1]. FHV RNA1 encodes protein A, which is the RNA-dependent RNA polymerase (RdRP) [2], and RNA2 encodes protein α, which is the capsid precursor protein and undergoes autocatalytic maturation cleavage into two viral capsid proteins [3]. Moreover, a subgenomic RNA3 (sgRNA3) is synthesized during RNA1 replication from the 3′ terminus of RNA1, and contains two open reading frames, which encode nonstructural proteins B1 and B2. While the function of protein B1 is unknown yet, FHV B2 functions as the viral suppressor of RNA silencing (VSR) to evade host RNA interference (RNAi) antiviral immunity [4], . Although the original host of FHV is grass grub, this virus can efficiently infect *Drosophila melanogaster*, the model insect, and can also abundantly replicate in mammalian [10], plant [11], and yeast cells [9], [12], [13]. Due to its wide range of hosts, simple genomic organization, as well as the presence of a VSR, FHV has become the most extensively studied member of *Nodaviridae*, and an attractive experimental model for the studies of various aspects of RNA virology, including viral RNA replication, assembly and structure, evolution, and the RNAi-based antiviral immunity [1], [6], [14], [15], [16], [17], [18], [19].

(+)-RNA viruses encode their own RdRPs, usually in concert with other viral and host factors, to catalyze RNA-templated viral RNA synthesis [20], [21]. The RNA replication of FHV is highly parallel with that of other (+)-RNA viruses. On the other hand, FHV as well as other nodaviruses encode only one RNA replicase protein, protein A, for viral RNA replication [10], [12]. These features make FHV an ideal and simplified model for studying viral RNA replication [10]. Previous studies have revealed that FHV protein A contains the conserved glycine-aspartate-aspartate (GDD) box that is present in all nodaviruses and strictly required for RNA replication by all known RdRPs of (+)-RNA viruses [22]. It has also been reported that FHV protein A replicates viral RNA in concert with the mitochondrial outer membrane and other viral or cellular factors [23], [24], [25], [26], [27], and mediates the formation of viral RNA replication complexes and small spherules by inducing membrane rearrangement [24], [26].

For viral RNA synthesis, a proper initiation is required for the integrity of viral RNA genomes and the efficiency of RNA synthesis [28], [29], [30]. So far, viral RdRPs have been reported to employ two principally distinct mechanisms for RNA synthesis initiation [31], [32], including primer-dependent and primer-independent (*de novo*) initiation. The primer-dependent initiation of RNA synthesis requires the involvement of either an oligonucleotide or a protein primer [33], [34], [35], [36], while the *de novo* initiation does not require primer, but uses the starting nucleotide to provide the 3′ hydroxyl group (3′-OH) for adding the next nucleotide. In the case of *de novo* initiation, RNA synthesis can be initiated at the terminal or an internal site of the template RNA [28], [29], [31], [37], [38]. However, although the RNA replication of FHV has been extensively studied, the mechanism employed by FHV protein A to initiate RNA synthesis has not been determined. Given the importance of RNA synthesis initiation for viral replication, this lack of knowledge hinders our understanding of FHV RNA replication as well as the use of FHV as the model to study viral RNA replication.

For this issue, the previous studies of Wuhan nodavirus (WhNV), another member of *Nodaviridae* originally isolated from cabbage fly (*Pieris rapae*) by our group [39], may provide some hints. WhNV protein A is also the RdRP [4], [11], [39], [40], and associates with the mitochondrial outer membrane during viral RNA replication [40]. A recent report by our group has revealed that WhNV protein A can initiate RNA synthesis via a *de novo* mechanism. Moreover, we found that WhNV protein A possesses a terminal nucleotidyl transferase (TNTase) activity, which can function to restore one or two 3′-proximal nucleotides of template RNA as a potential mechanism for rescuing 3′-terminal nucleotide loss [41]. However, previous phylogenetic analysis of WhNV indicates that WhNV is evolutionarily distant with alphanodaviruses such as FHV [4], [11]; moreover, it has been reported that internal initiation, instead of the proposed premature termination mechanism for FHV [42], is the mechanism governing the sgRNA3 synthesis of WhNV [11]. Thus, we cannot simply assume that the findings of WhNV apply to FHV in every aspect, and it is intriguing to ask whether FHV protein A initiate RNA synthesis in a similar manner, and also possesses a TNTase activity.

## Materials and Methods

### Plasmid Construction, and Recombinant Protein Expression and Purification

To construct the bacterial expression vector for MBP-Pro A or its derivative, the FHV protein A coding sequence was PCR amplified from the cDNA of the FHV RNA1 genome plasmids FHV1(1, 0) [43], [44] with the primers Pro A-For (GCGAATTCA TGACTCTAAAAGTTATTCTTGGA) and Pro A-Rev (GCGTCGACTCACTTC CGGTTGTTGGAAGGCTGT). The *Eco*R I and *Sal* I nuclease sites were introduced during PCR amplification. The polymerase active site mutagenesis GDD to GAA, containing the double substitution of both Asp692 and Asp693 to alanine, was also carried out by PCR-mediated mutagenesis [45] (Primers: TTTAATCGGACC GAAGTGCGGTGCCGCCGGTCTTTCCCGGGC and TTTGAATGATAGCCCGG GAAAGACCGGCGGCACCGCACTTCGGTC). After digested with *Eco*R I and *Sal* I (Takara Bio, Shiga, Japan), the PCR amplified fragments were cloned into the *Eco*R I -*Sal* I site of the pMAL-c2X vector (New England Biolabs, Ipswich, MA). Transformants were analyzed by restriction enzyme mapping and confirmed by DNA sequencing (BioDev-Tech. Co, Beijing, China). All the primers used were purchased from Invitrogen (Carlsbad, CA).

The recombinant protein MBP-Pro A, its mutant and MBP alone were expressed and purified as previously described [41]. Proteins were confirmed by 10% SDS-PAGE and Western blots. Protein concentrations were quantified with Bio-Rad Quantity One software using known amounts of bovine serum albumin as controls.

### Construction and Purification of RNA Templates

The DNA fragment of the 3′-end of the (+)RNA1, (–)RNA1 was amplified from the cDNA of the FHV RNA1 genome plasmids FHV1(1, 0). Following the manufacturer’s instructions (Promega, Madison, WI), each pair of the primers was designed to include one primer, which contains a portion of 5′-end of expected RNA product plus the T7 promoter region (TAATACGACTCACTATAGg), and the other primer whose sequence is reverse-complementary to the 3′-end of the expected RNA product. RNA was transcribed *in vitro* from PCR products using T7 RNA polymerase (Promega, Madison, WI) for 4 h. DNA templates were removed by RQ1 RNase-free DNase I (Promega) at 37°C for 1 h. After that, TRIzol reagent (Invitrogen) was used to extract the RNAs according to the manufacturer’s protocol. The blocking of RNA template was conducted as previously described [41].

To get rid of T7 RNA polymerase and NTP, all the RNA templates were electrophoresed in a 6% polyacrylamide–7 M urea gel and further purified by Poly-Gel RNA Extraction Kit (Omega bio-tek, Norcross, GA) according to the manufacturer’s instruction. Purified RNAs were quantified with Bio-Rad Quantity One using known amounts of RNA as controls after Northern blots.

### RdRP Activity Assay

Standard RdRP reaction mixtures (total volume 15 µL) contained 25 mM HEPES (pH 8.0), 1 mM MnCl_2_, 100 mM NaCl, 4 mM DTT, 250 µM DIG RNA Labelling Mix (Roche, Basel, Switzerland), 40 units of RNasin (Promega), 0.3 µg template RNA (∼100 nM) and 500 nM purified MBP-Pro A or its mutant. Reactions were incubated at 30°C for 90 min. The reaction mixture was supplemented added with isopyknic RNA loading mix [20% 10×loading buffer (Takara Bio), 50% formamide and 20% formaldehyde] and then incubated at 65°C for 5 min. The 10×loading buffer contains 1% SDS, 50% glycerol and 0.05% bromophenol blue. Then, the products were analyzed by electrophoresis on 1.8% agarose-formaldehydegel denaturingn gels and transferred to N+ nylon membranes (Millipore) and fixed. The transferred nylon membrane was incubated with anti-DIG-AP antibody (Roche), and then incubated with CDP-Star™ (Roche), a kind of chemi-luminescent alkaline phosphatase substrates, at 37°C for 10–15 minutes. The signal is then visualized by radiography on an X-ray film. The RNAs were quantified with Bio-Rad Quantity One software.

Gliotoxin were purchased from EMD Chemicals (Billerica, MA), and phosphonoacetic acid (PAA) were purchased from Sigma (St Louis, MO). Gliotoxin or PAA was supplemented into the standard RdRP reaction mixtures. To explore the effects of primers in the RdRP reaction, the specific RNA primer (GUUUUCGAAACAAAU ) were synthesized by Takara Bio.

All reactions were stopped by adding the RNA loading mix, and analyzed by Northern blots as previously described [41]. The sequences of 1–100 nucleotides at the 5′-end of RNA1(+) (Probe 1) hybridize the template RNA when 3′-end 1–201 nucleotides of RNA1(−) was used as template. Similarly, the sequences of 1–100 nucleotides at the 5′-end of RNA1(−) (Probe 2) and 1–24 nucleotides at the 3′-end of RNA1(+) (Probe 3) (GUUCUAGCCCGAAAGGGCAG AGGU) were used as the probes separately hybridize the template RNA and the RdRp products when 3′-end 1–191 nucleotides of RNA1(+) was used as template. All probes were labeled with digoxigenin (DIG)-UTP (Roche, Basel, Switzerland) by *in vitro* transcription according to our standard procedures [4], [11].

### Reverse Transcription (RT) Polymerase Chain Reaction (RT-PCR) Analysis

Purified RNAs extracted from RdRP reaction mixtures was subjected to RT with M-MLV (Promega) according to the manufacturer’s protocol. The strand-specific oligodeoxynucleotide primers (+)3′RNA1-For (GGAGAAACCC AGCGTGGTGGCATAC) and (−)3′RNA1-Rev (ACTCGGTTCCCTCCACTCTGA GGA), respectively associated with the synthesized plus- and minus-strand RNAs, were used for RT-catalyzed cDNA synthesis, followed by PCR amplification of cDNAs. PCR was performed with (+)3′RNA1-For/(+)3′RNA1-Rev (ACCTCTGCCCTTTCGGGCTAGAACGGGT) or (−)3′RNA1-For (GTTTTCGAA ACAAATAAAACAG)/(−)3′RNA1-Rev primers for amplification of cDNAs. The RT reactions in the absence of any primer were set as controls. Results were detected using UV transillumination after ethidium bromide staining. The cDNAs were then cloned into pMD-18T vector (Takara Bio), followed by DNA sequencing (BioDev-Tech. Co).

### TNTase Activity Assay

The TNTase reaction was performed as previously described [41]. In brief, 250 µM DIG-labeled UTP mix (65% DIG-labeled UTP together with 35% cold UTP) was used instead of 250 µM DIG RNA Labelling Mix in standard RdRP reaction, and other components were similar with standard RdRP reaction. Other NTPs in concentration of 250 µM were supplemented into the standard TNTase reaction mixtures as indicated. TNTase reaction products were transferred onto N+ nylon membrane (Millipore), followed by the treatment with anti-DIG-AP antibody (Roche). Results were detected by radiography using the DIG RNA detection kit (Roche) according to the manufacturer’s protocol.

## Results

### FHV Protein A Possesses RdRP Activity in the Absence of an Exogenous Primer

**Figure 1:**
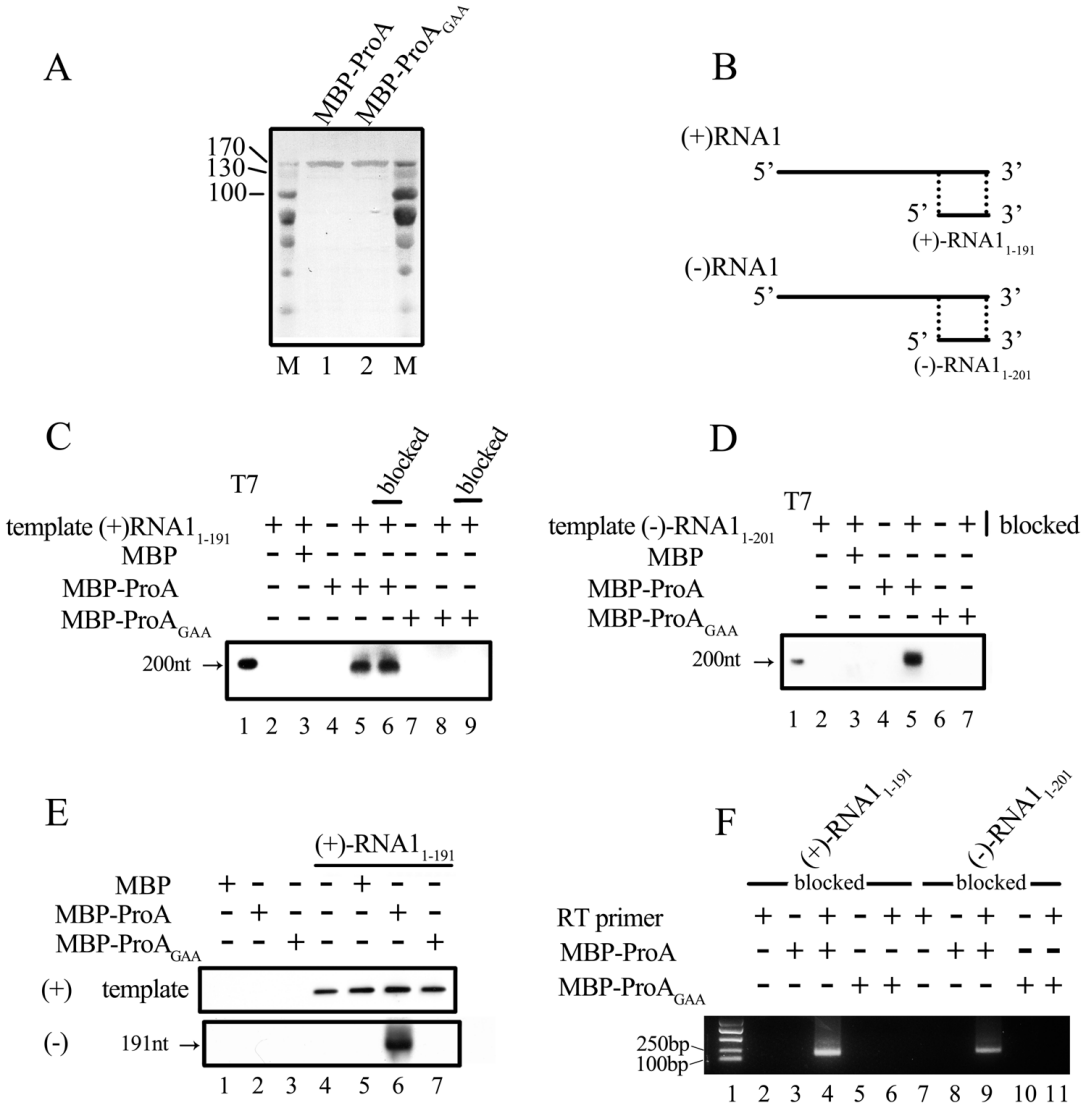
FHV protein A possesses RdRP activity. (A) Electrophoresis analysis of purified MBP-Pro A and its mutants. Lane M, molecular weight markers (in kDa); Lane 1, MBP-Pro A; Lane 2, MBP-Pro A_GAA_, the MBP fusion GDD-to-GAA mutant protein A. (B) Schematic of the RNA templates used for RdRP assays. (C) The indicated template, intact or with its 3′ end blocked via oxidation, was incubated with the indicated proteins and DIG RNA Labeling mix. The reaction products were by electrophoresis on a denaturing formaldehyde-agarose gel and detected.Lane 1, synthesized DIG-labeled RNA at the designated size (200 nt) generated by T7 polymerase-mediated *in vitro* transcription (D) The blocked template (−)-RNA1_1–201_ was incubated with the indicated proteins. Templates and reaction products were analyzed and detected as in (C). (E) The blocked template (+)-RNA1_1–191_ was incubated with the indicated proteins. Templates and reaction products were analyzed on denaturing formaldehyde-agarose gel and detected via Northern blot analysis using the DIG-labeled probes 2 and DIG-labeled probes 3 (GUUCUAGCCCGAAAGGGCAGAGGU). (F) The RNA products synthesized in (C) and (D) were subjected to RT-PCR. Reverse transcription was conducted in the presence or absence of specific RT primers, followed by PCR amplification. PCR products were electrophoresed through 1.0% agarose gel and visualized by ethidium bromide staining. Lane 1, DNA ladder.

To study the activity of FHV protein A, we first expressed wild-type and replication-defective mutant forms of soluble recombinant protein A as MBP-fusion proteins in *E. coli*, and purified the fusion proteins using amylose resins. The replication-defective mutant protein A was generated by mutating the conserved RNA replication GDD motif to GAA (MBP-Pro A_GAA_), which would normally disrupt the RdRP activity [22], . As shown in Figure 1A, both MBP-Pro A and MBP-Pro A_GAA_ were efficiently expressed at expected molecular weights (∼155 kDa).

To examine if FHV protein A could initiate the synthesis of FHV RNA1-specific sequences *in vitro*, we used the 3′ end 191 nucleotides of (+)-RNA1 and 201 nucleotides (−)-RNA1, indicating as (+)-RNA1_1–191_ and (−)-RNA1_1–201_, as the templates for RNA synthesis (Figure 1B). Besides, although we did not determine if FHV protein A also possesses a TNTase activity, to exclude the possible interference of the potential TNTase activity of FHV protein A, we blocked the 3′-ends of RNA templates, in which the 3′-OHs were oxidized. The oxidized 3′-OH cannot be used to add extra nucleotides for oligonucleotide elongation. The RNA products were synthesized by protein A in the reaction mixture containing DIG-labeled UTP together with other NTPs (DIG RNA labeling mixture), and then subjected to denaturing formaldehyde-agarose gel electrophoresis, followed by detecting DIG signals with anti-DIG-AP antibody. As shown in Figure 1C, MBP-Pro A efficiently initiated (−)-RNA1 synthesis from the (+)-RNA1_1–191_ template, no matter if its 3′-end was blocked or unblocked (lanes 5 and 6), while the negative controls, MBP alone and MBP-Pro A_GAA_, could not initiate RNA synthesis (lanes 3, 8, and 9). Similar results were obtained when the (−)-RNA1_1–201_ template was used for (+)-RNA synthesis (Figure 1D). These results show that purified recombinant FHV protein A can initiate RNA synthesis in the absence of an exogenous primer and other viral or cellular factors.

To further confirm that the RNA products were synthesized from the provided template, the 250 µM NTP mixture was used instead of 250 µM DIG RNA labeling mixture (containing DIG-UTP) in the standard RdRP reaction, and the RNA products were then detected via Northern blots using DIG-labeled probes specific for FHV (+)-RNA1 or (−)-RNA1 (described in Materials and Methods). As shown in Figure 1E, the (−)-RNA product could be detected (lane 6). Moreover, the RNA products were reverse transcribed into DNA fragments at the expected size (∼200 nt) when specific RT-PCR primers were used (Figure 1F, lanes 4 and 9). On the other hand, when specific primers were not supplemented in the reverse transcription step of RT-PCR, DNA fragments were not detected (Figure 1F, lanes 3 and 8), thereby excluding the possibility that some DNA contaminants existed in the purified RNA1 templates. Moreover, these reverse transcribed DNA fragments were further subjected to DNA sequencing analyses, which confirmed that the RNA products were synthesized specifically from the provided RNA templates (data not shown). Of note, these experiments have been independently repeated at least three times.

Taken together, our results show that FHV protein A possesses the RdRP activity to initiate the RNA synthesis from RNA1-specific templates in the absence of an exogenous primer.

### FHV Protein A Initiates RNA Synthesis via a *de novo* Mechanism

**Figure 2:**
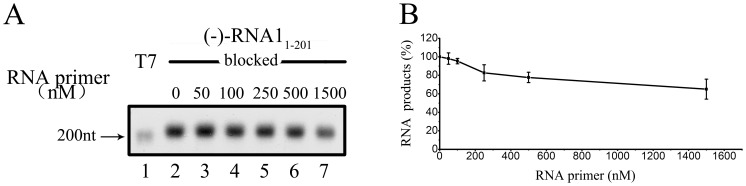
FHV protein A initiates RNA synthesis via a *de novo* mechanism. (A) The template, (−)-RNA1_1–201_ with its 3′-end blocked, was incubated with the MBP-Pro A and different concentrations specific RNA primer. Reaction products were analyzed on denaturing formaldehyde-agarose gel and detected as described in “Materials and Methods”. Lane 1, synthesized DIG-labeled RNA at the designated size (200 nt) generated by T7 polymerase-mediated *in vitro* transcription. (B) The synthesized RNA products from the experiments in (A) were measured via Bio-Rad Quantity One software, and the relative RdRP activities were determined by comparing the RNA product level in the presence of the indicated concentration of primer with the RNA product level without the primer. Error bars represent the standard deviation (S.D.) values from at least three independently repeated experiments.

After determining that FHV protein A possesses the RdRP activity in the absence of an exogenous primer, we sought to examine if *de novo* initiation is the mechanism by which FHV protein A initiates RNA synthesis. To this end, (−)-RNA1_1–201_ with its 3′-end being blocked (oxidized) was used as the template, and the RNA primer specific to 3′-end of (−)RNA1 was added to the RdRP reaction at different concentrations (50 nM-1.5 µM) to determine if the RNA primer could promote (+)-RNA synthesis. As shown in Figure 2, the presence of exogenous RNA primer exhibited no promoting, but minimal inhibitory effect to the (+)-RNA synthesis, further confirming that the RdRP-catalyzed RNA synthesis does not require an exogenous primer.

In the absence of an exogenous primer, some RNA template can use its 3′-terminal sequence to fold back and form a hairpin structure. The folded back sequence can serve as an endogenous primer to initiate RNA synthesis, and this mechanism is called “copy-back” initiation, which is also primer dependent [33], [34], [37]. If such a mechanism is used, we should detect the product with the double size of the RNA template, as the synthesized strand is covalently linked to the template via the hairpin. Our previous results in Figures 1C and E showed no double-sized product (∼400 nt). Moreover, our results showed that the templates with oxidized 3′-OH exhibited the same ability for RNA synthesis initiation (Figure 1C, lanes 5 vs. 6), thereby excluding the possibility that FHV protein A uses the copy-back mechanism. Taken together, our results show that FHV protein A initiates RNA synthesis generally via a *de novo* mechanism.

### Biochemical Characterization of the RdRP Activity of FHV Protein A

**Figure 3:**
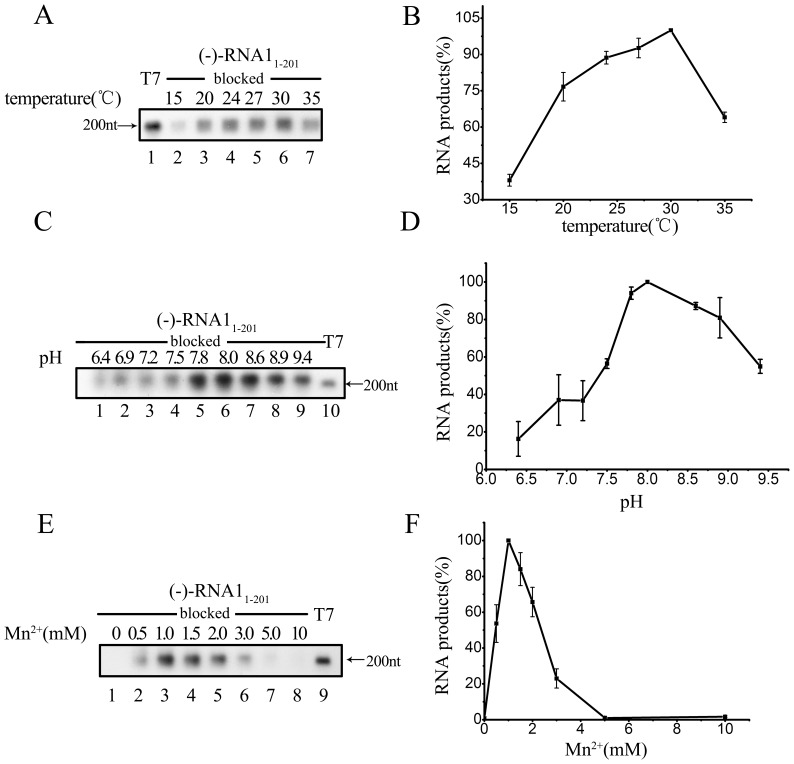
Effects of different reaction conditions on *de novo* RNA synthesis. (A) The template, (−)-RNA1_1–201_ with its 3′-end blocked, was incubated with the MBP-Pro A at different temperature. Reaction products were analyzed on denaturing formaldehyde-agarose gel and detected as described in “Materials and Methods”. Lane 1, synthesized DIG-labeled RNA at the designated size (200 nt) generated by T7 polymerase-mediated *in vitro* transcription. (B) The synthesized RNA products from the experiments in (A) were measured via Bio-Rad Quantity One software Error bars represent the standard deviation (S.D.) values from at least three independently repeated experiments. (C) The template (−)-RNA1_1–201_ with its 3′-end blocked, was incubated with the MBP-Pro A at the different pH. Reaction products were analyzed on denaturing formaldehyde-agarose gel and detected as described in “Materials and Methods”. Lane 1, synthesized DIG-labeled RNA at the designated size (200 nt) generated by T7 polymerase-mediated *in vitro* transcription. (D) The synthesized RNA products from the experiments in (C) were measured via Bio-Rad Quantity One software Error bars represent the standard deviation (S.D.) values from at least three independently repeated experiments. (E) The template, (−)-RNA1_1–201_ with its 3′-end blocked, was incubated with the MBP-Pro A and different concentrations of Mn^2+^. Reaction products were analyzed on denaturing formaldehyde-agarose gel and detected as described in “Materials and Methods”. Lane 1, synthesized DIG-labeled RNA at the designated size (200 nt) generated by T7 polymerase-mediated *in vitro* transcription. (F) The synthesized RNA products from the experiments in (E) were measured via Bio-Rad Quantity One software Error bars represent the standard deviation (S.D.) values from at least three independently repeated experiments.

To characterize the biochemical properties of FHV protein A, we examined its RdRP activity under different reaction conditions, such as temperature, divalent metallic ion concentration, and pH. Because FHV protein A initiates RNA synthesis via a *de novo* mechanism, we determined the optimal conditions for the *de novo* RNA synthesis using (−)-RNA1_1–201_ as the template. Our results showed that the RdRP activity of FHV protein A exhibited the highest activity at ∼30°C (Figure 3A and B), and favored a mildly basic pH at 8.0. Moreover, similarly with WhNV protein A, the RdRP activity of FHV protein A requires the presence of Mn^2+^. The highest RdRP activity was observed at 1 mM, while higher concentrations of Mn^2+^ negatively affected the RNA synthesis (Figure 3E and F).

**Figure 4:**
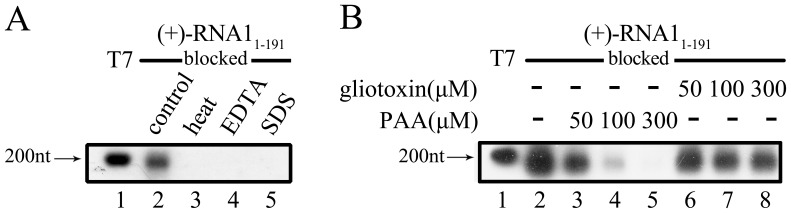
Effects of RdRP inhibitors. (A) The blocked (+)-RNA1_1–191_ template was reacted with MBP-Pro A under the presence or absence of indicated conditions, such as standard condition (lane 2), heat at 95°C for 2 min (lane 3), 20 mM EDTA (lane 4), or 0.6% SDS (lane 5). Lane 1, synthesized DIG-labeled RNA at the designated size (200 nt) generated by T7 polymerase-mediated *in vitro* transcription. (B) The blocked (+)-RNA1_1–191_ template was reacted with MBP-Pro A in the presence of RdRP inhibitor PAA (lanes 3–5) or gliotoxin (lanes 6–8) at the indicated concentrations. Lane 1, DIG-labeled RNA at the designated size (200 nt) generated by T7 polymerase. The reaction products were analyzed on denaturing formaldehyde-agarose gel and detected as described in “Materials and Methods”.

Moreover, we examined the responses of the RdRP activity to heat, EDTA, and SDS. Our results showed that any one of them almost abolished the RdRP-catalyzed RNA synthesis (Figure 4A). Last, we examined the effects of RdRP-specific inhibitors on FHV protein A. Gliotoxin and phosphonoacetic acid (PAA) are well-known inhibitors to some virus-encoded RdRPs [49], [50], [51], [52], [53], [54], [55], [56], and we added gliotoxin or PAA into the RdRP reaction. As shown in Figure 4B, PAA dramatically inhibited the RdRP activity of FHV protein A in a dose-response manner (lanes 2–5); on the other hand, gliotoxin exhibited minor inhibition to FHV protein A (lanes 6–8). These experiments have also been conducted using (−)-stranded templates, and similar results were obtained (data not shown). Of note, the sensitivity of FHV protein A RdRP activity to PAA instead of gliotoxin is similar with that of WhNV protein A [41].

### FHV Protein A Possesses a TNTase Activity

**Figure 5:**
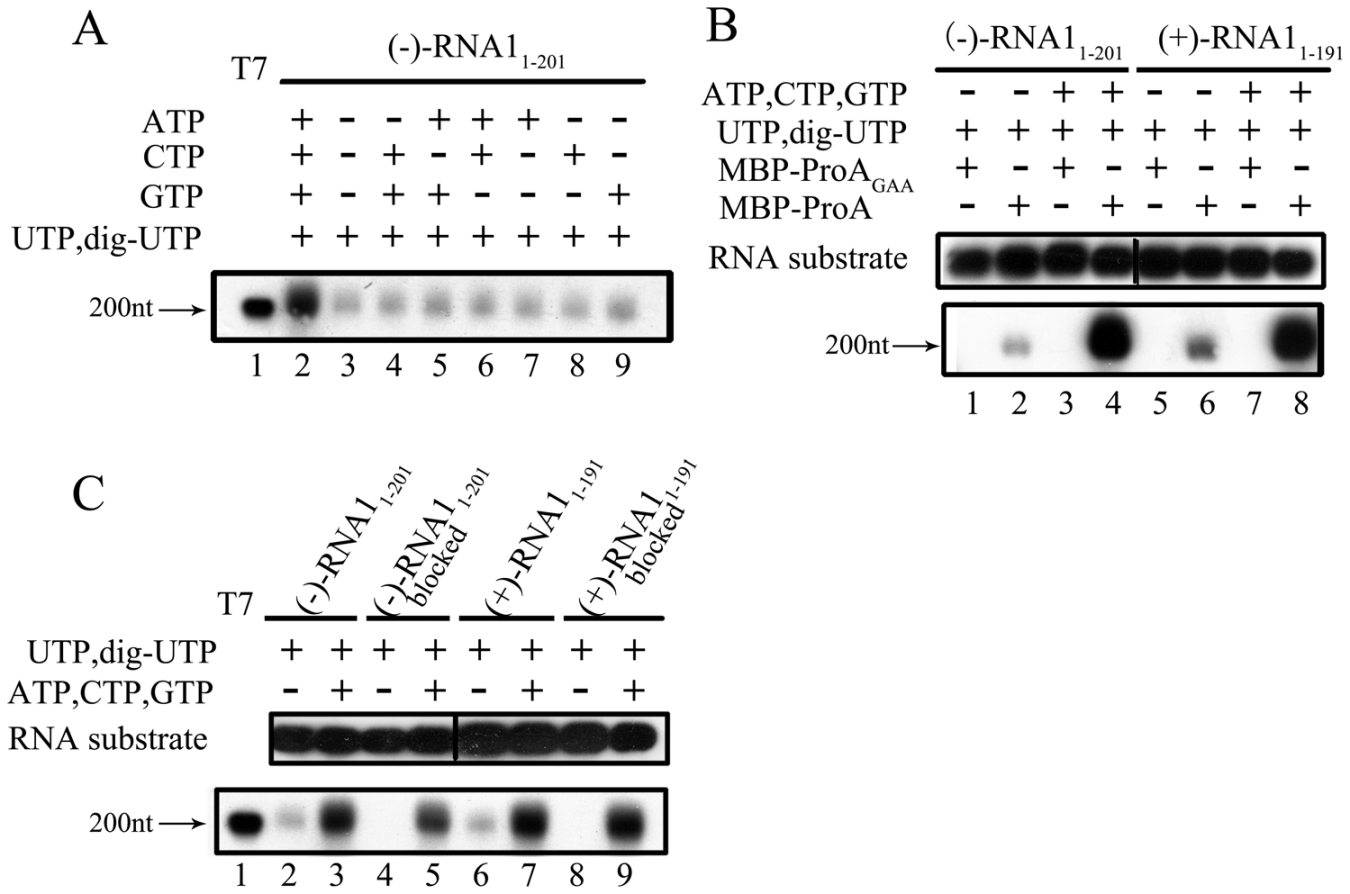
FHV protein A possesses TNTase activity. (A) The (−)-RNA1_1–201_ substrate was reacted with MBP-Pro A with DIG-labeled UTP mix (65% DIG-labeled UTP together with 35% UTP) in the absence (lane 3) or presence of indicated NTPs (lanes 2, 4–9). (B) The (−)-RNA1_1–201_ (lanes 1–4) or (+)-RNA1_1–191_ (lanes 5–8) substrates as well as DIG-labeled UTP mix were reacted with MBP-Pro A or MBP-Pro A_GAA_ as indicated, in the absence (lanes 1, 2, 5, and 6) or presence (lanes 3, 4, 7 and 8) of ATP, CTP, and GTP mix. (C) The (−)-RNA1_1–201_ (lanes 2–5) or (+)-RNA1_1–191_ (lanes 6–9) substrates were intact (lanes 2, 3, 6 and 7) or 3′-end blocked by oxidation (lanes 4, 5, 8 and 9). The indicated substrates were incubated with DIG-labeled UTP mix in the presence or absence of ATP, CTP, and GTP mix. For (A–C), the substrates and TNTase reaction products were analyzed and detected as described in “Materials and Methods”.

TNTase activity has been found in several viral RNA polymerases to add nucleotides to the 3′ end of RNAs [33], [57], [58], [59], including WhNV protein A [41]. To determine if FHV protein A also contains TNTase activity, we incubated MBP-Pro A with (−)-RNA1_1–201_, whose 3′-end was not blocked or oxidized, in the mixture containing DIG-labeled UTP mix (65% DIG-labeled UTP together with 35% non-labeled UTP) and/or other NTPs. As shown in Figure 5A, recombinant FHV protein A exhibited the TNTase activity to add DIG-labeled UTP to the 3′ end of the (−)-RNA1 substrate in the presence of indicated NTPs (lanes 2–9). It is worthy to note that in the presence of all NTPs, protein A possessed both RdRP and TNTase activities, resulting in much higher level of DIG-labeled products (Figure 5A, lane 2). Moreover, since the presence of one or two other NTPs did not interfere with the signal of DIG-UTP addition, the TNTase activity of FHV protein A may have some preference for UTP.

It has been previously reported that for RdRPs that also possess TNTase activity, the GDD motif is required for both activities [58]. Our results showed that using either (−)-RNA1_1–201_ or (+)-RNA1_1–191_ as the substrate/template, the GDD-to-GAA mutation (MBP-Pro A_GAA_) resulted in the complete loss of both TNTase (Figure 5B, lanes 1 and 5) and RdRP (Figure 5B, lanes 3 and 7) activities of FHV protein A, further confirming that the GDD motif is required for both activities of protein A, and also eliminating the possibility that some unknown cellular TNTase contaminated the purified MBP-Pro A preparation.

The TNTase-catalyzed addition of nucleotide to the 3′-end of RNA requires the presence of 3′-OHs in RNA substrates. To determine if this also applies to the TNTase activity of FHV protein A, we examined the TNTase reaction using the RNA substrates, (−)-RNA1_1–201_ and (+)-RNA1_1–191_, in the presence or absence of 3′-OH oxidation. As shown in Figure 5C, when the 3′-OHs of the RNA substrates were oxidized, protein A lost its ability to add DIG-UTP to the substrates (lane 2 vs. 4, and lane 6 vs. 8). On the other hand, FHV protein A still exhibited the RdRP activity to catalyze RNA synthesis in the presence or absence of the oxidation of 3′-OHs (Figure 5C, lanes 3, 5, 7, and 9). Of note, all these experiments have been independently repeated at least three times. Altogether, our data demonstrate that FHV protein A possesses the TNTase activity to add extra nucleotides to the 3′ end of (+)-or (−)-stranded RNA substrate.

### The RdRP Activity of FHV Protein A Relies on the 3′-proximal Nucleotides of RNA1

**Figure 6:**
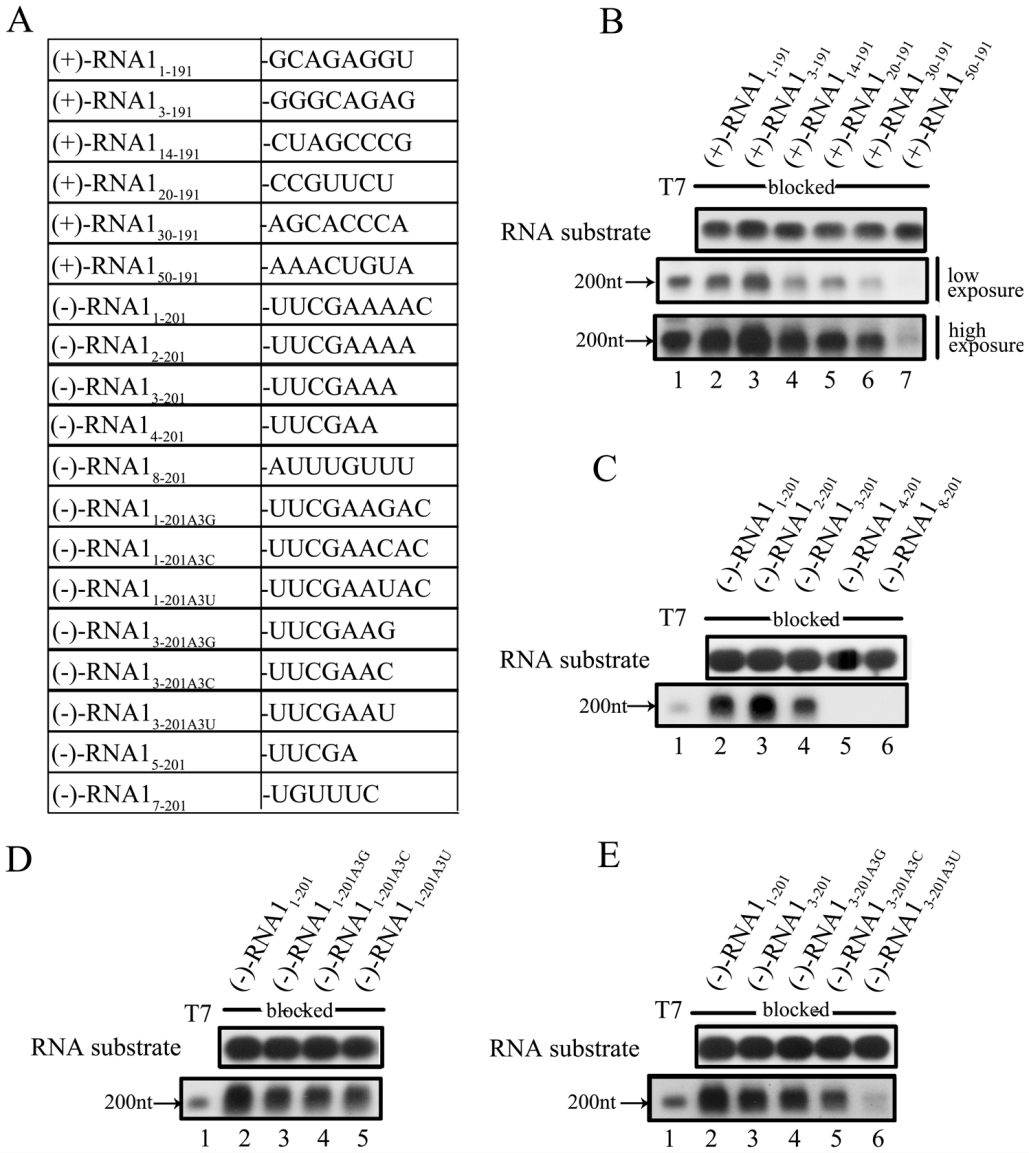
The RdRP activities of protein A depend on the 3′-proximal nucleotides of RNA1. (A) The (−)RNA and (+)RNA templates/substrates with different 3′-end sequences are shown. (B) The 3′-OH blocked (+)RNA templates with 3′ -proximal deletion of 0 to 49 nucleotides (lanes 2–7) were reacted with MBP-Pro A as indicated. The templates and RdRP reaction products were analyzed on denaturing formaldehyde-agarose gel and detected as described in “Materials and Methods”. (C) The 3′-OH blocked (−)RNA substrates with 3′-proximal deletion of 0 to 7 nucleotides (lanes 2–6) were reacted with MBP-Pro A as indicated. The substrates and reaction products were analyzed and detected as described in “Materials and Methods”. (D) The blocked (−)-RNA1_1–201A3G,_ (−)-RNA1_1–201A3C_ and (−)-RNA1_1–201A3U_ were reacted with MBP-Pro A. Templates and reaction products were analyzed and detected as described in “Materials and Methods”. (E) The blocked (−)-RNA1_3–201A3G_, (−)-RNA1_3–201A3C_ and (−)-RNA1_3–201A3U_ templates were reacted with MBP-Pro A. Templates and reaction products were analyzed and detected as described in “Materials and Methods”. For (B-E), lane 1 represents synthesized DIG-labeled RNA at the designated size (200 nt) generated by T7 polymerase-mediated *in vitro* transcription.

It has been previously reported that the 3′ region of RNA template is critical for viral RdRP activity [28], [29], [30], [48], . To determine if this also applies to FHV protein A, we evaluated the impacts of mutations or deletions at the 3′ ends of (−)-RNA1_1–201_ and (+)-RNA1_1–191_ on RNA synthesis (Figure 6A). To exclude the interference of the TNTase activity, the templates were blocked by oxidizing the 3′-OHs.

We first evaluated the effects of 3′ nucleotide loss of (+)-RNA1_1–191_ on RdRP-catalyzed (−)-RNA synthesis. The RNA synthesis initiation could tolerate the loss of two nucleotides at the 3′ end (Figure 6B, lanes 2 and 3), while more nucleotide loss (13–29 nts) inhibited the RNA synthesis (Figure 6B, upper panel, lanes 4–6). Moreover, when 49 nucleotides were lost from the 3′ end of the (+)-RNA1_1–191_ template, the RdRP reaction was mostly abolished (Figure 6B, under panel, lane 7), indicating that the 30–50 nucleotides of the 3′-end of (+)-RNA1 template are critical for the synthesis initiation of (−)-RNA.

Next, we evaluated the effects of 3′ nucleotide loss of (−)-RNA1_1–201_ on the RdRP-catalyzed (+)-RNA synthesis. As shown in Figure 6C, the RNA synthesis initiation tolerated one-nucleotide loss at the 3′ end (lane 3), while losing two nucleotides resulted in a decrease of RNA synthesis (lane 4). More dramatically, when three or more nucleotides were lost, the RdRP reaction was completely blocked (Figure 6C, lanes 5 and 6). These data indicate that the 3 nucleotides of the 3′-end of (−)-RNA1 template are required for the synthesis initiation of (+)-RNA. The differential tolerance of FHV protein A to the 3′-end loss of (+)- and (−)-RNA1 templates suggest that FHV protein A exhibits some sequence specificity to the 3′-end regions of the (+)- and (−)-strand RNA1 templates, and probably uses distinct strategies to initiate RNA synthesis on the different templates.

The 5′-terminal sequences of FHV RNA1, RNA2, and sgRNA3 are GUUUUC, GUAAAC, and GUUACC. Correspondingly, the 3′-terminal sequences of FHV (−)-stranded RNA1, RNA2, and sgRNA3 are 5′-GAAAAC-3′, 5′-GUUUAC-3′, and 5′-GGUAAC-3′, respectively. Previous study has already reported that the U at the position 2 of FHV RNA2 is essential for its replication [44]. Our above study also revealed that the 3′-end three nucleotides of (−)-RNA1, which corresponds to the 5′-end three nucleotides of (+)-RNA1, are required for the RNA synthesis initiation. Thus, it is interesting to investigate the impact of the A at the 3′-end position 3 of FHV (−)-RNA1 on RNA1 replication. To this end, we generated the (−)-RNA1_1–201_ template with the A at the position 3 being mutated to G, C, or U, which was indicated as (−)-RNA1_1–201A3G_, (−)-RNA1_1–201A3C_, or (−)-RNA1_1–201A3U_, respectively. Our results showed that these mutations at the (−)-RNA1_1–201_ template only resulted in minor reduction of the RNA synthesis (Figure 6D, lanes 2–5). Previously, we have shown that the 3′-end three nucleotides of (−)-RNA1 are required for the synthesis initiation. Thus, we also examined the effects of these mutations at the (−)-RNA1_3–201_ template, whose 3′-end two nucleotides are deleted. Our results showed that the A3G and A3C mutations at the (−)-RNA1_3–201_ template resulted in minor reduction of the RNA synthesis (Figure 6E, lanes 3–5), but the A3U mutation dramatically reduced the RNA synthesis (Figure 6E, lane 6). Of note, all the experiments have been independently repeated.

### The TNTase Activity of FHV Protein A can Function to Repair the 3′-end Nucleotide Loss of the Template and Rescue Synthesis Initiation *in vitro*

Since the RdRP and TNTase activities of a given viral RNA polymerase share the same catalytic motif (GDD), it was very hard to dissect the two activities and study the role of one activity in the absence of the other. To solve this issue, we have designed a set of experiments in our previous study [41], which could help determine if the TNTase activity of FHV protein A can function to restore the replicability of template with 3′-terminal nucleotide(s) loss.

**Figure 7:**
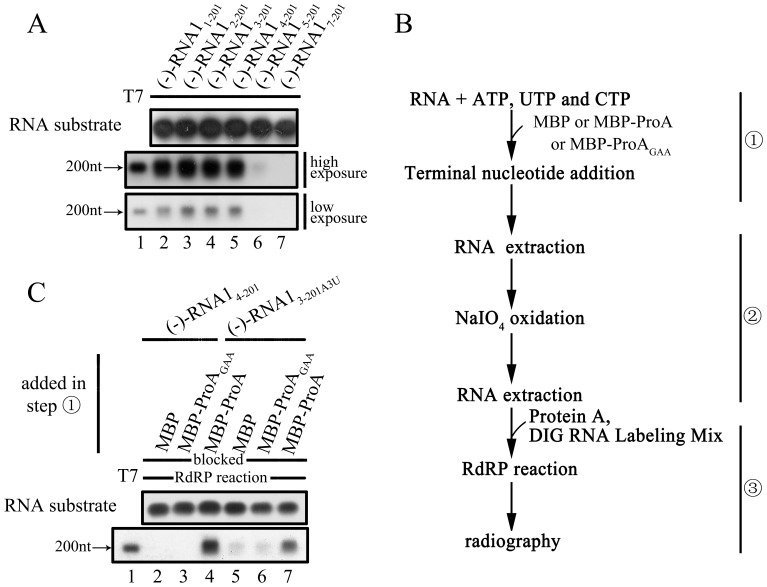
The TNTase activity of protein A can restore the replication competency of the templates with 3′-terminal nucleotide deletion. (A) The (−)RNA substrates with 3′-proximal deletion of 0 to 6 nucleotides (lanes 2–7) were reacted with MBP-Pro A and DIG-labeled UTP mix as indicated. The substrates and reaction products were analyzed and detected as described in “Materials and Methods”. (B) Schematic of the experimental procedures used in (C). (C) The RNA templates, (−)-RNA1_4–201_ (lane2–4) and (−)-RNA1_3–201A3U_ (lane5–7) were reacted with indicated protein. Templates and reaction products were analyzed and detected as described in “Materials and Methods”. For (A,C), lane 1 represents synthesized DIG-labeled RNA at the designated size (200 nt) generated by T7 polymerase-mediated *in vitro* transcription.

Firstly, we evaluated the effects of 3′ nucleotide loss of (−)-RNA1_1–201_ on the TNTase-mediated nucleotide addition. When one, two or three nucleotides were lost at the 3′ end of the (−)-stranded substrate, the nucleotide addition could be detected using a standard TNTase assay (Figure 7A, lower panel, lanes 2–5). However, when ≥4 nucleotides were lost, the TNTase reaction was almost abolished (Figure 7A, upper panel, lanes 6–7). These results indicate that the TNTase activity of FHV protein A can restore the loss of 1–3 nucleotides in the template.

Furthermore, we designed a set of experiments to deliberately separate the TNTase and RdRP reactions. As illustrated in Figure 7B, in the first step, we added ATP, UTP and CTP to allow the TNTase reaction of the indicated RNA substrates/templates; since GTP is absent, the RdRP reaction cannot happen here. Then, in the second step, the TNTase-reacted RNAs were purified, followed by the oxidation of their 3′-end OHs to block further TNTase reaction. Last, in the third step, the 3′-end oxidized templates were subjected to the RdRP reaction in the presence of all four NTPs. This set of experiments allows us to evaluate the potential terminal repair function of the TNTase activity without the interference of its RdRP activity. Our results showed that when protein A was supplemented in the TNTase reaction (the first step), the replicabilities of the reacted (−)RNA1 templates, (−)-RNA1_4–201_ and (−)-RNA1_3–201A3U_, were efficiently rescued (Figure 7C, lanes 2–3 vs. 4; lanes 5–6 vs. 7). These data have been independently repeated at least three times, and indicate that the TNTase activity of FHV protein A can function to rescue the replication of the RNA1 template by repairing the 3′-end nucleotide loss.

## Discussion

FHV is a well recognized model for studying RNA replication of (+)-RNA viruses. However, although many progresses have been achieved by studying FHV RNA replication, the mechanism employed by FHV replicase, protein A, to initiate RNA synthesis had not been determined before this study. Here, we uncovered that FHV protein A can initiate RNA synthesis via a *de novo* (primer-independent) mechanism. Moreover, we found that FHV protein A possesses the TNTase activity, which was able to restore the nucleotide loss at the 3′-end initiation site of RNA template to rescue RNA synthesis initiation *in vitro*.

An accurate initiation is required for the proper replication of viral RNA, and the mechanisms of RNA synthesis initiation by virus-encoded RdRPs are principally classified as two major types based on their dependence on the involvement of primers. The *de novo* initiation mechanism is primer-independent, and RdRPs do not require primer, but normally synthesize dinucleotides as the starting nucleotides on the 3′-end of RNA templates for adding next nucleotides [60], [61]. On the other hand, the primer-dependent initiation mechanism requires the involvement of a primer, which could be an oligonucleotide or a protein [33], [34], [35], [36]. For example, rabbit hemorrhagic disease virus and hepatitis C virus, both of which are (+)-RNA viruses, use RNA primers for the synthesis initiation of their RNA genome [33], [34], [35], while picornaviruses use a uridylylated form of the VPg protein (also named as nonstructural protein 3B) as primer for the synthesis of both (+)- and (−)-RNAs [36]. Moreover, for the primer-dependent initiation mechanism, the primer could not only be exogenous, but also endogenous. In this case, the 3′ end of some RNA templates can fold back to form a hairpin structure, which serves as the primer to initiate RNA synthesis; and this mechanism is also primer-dependent and named “copy-back” initiation [33], [34], [37]. For FHV, recombinant FHV protein A can initiate RNA synthesis in the absence of an exogenous primer or other viral or cellular factors, and the presence of RNA primer specific to 3′-end of (−)RNA1 did not promote the synthesis initiation. Moreover, the copy-back initiation mechanism was also excluded by using 3′-end blocked (3′-OH oxidized) RNA as template (Figure 1C). However, the possibility that a primer is involved in certain circumstances of FHV RNA replication cannot be completely excluded. Thus, we conclude that FHV protein A initiates RNA synthesis generally, if not only, via a *de novo* mechanism.

Our previous study of WhNV protein A has determined that this nodaviral replicase protein also initiates RNA synthesis *de novo* [41]. Moreover, these two protein As exhibited similar sensitivity to RdRP inhibitor PAA but not to gliotoxin [41] (Figure 4B). It is interesting that gliotoxin is normally considered as an inhibitor of the replication of (+)-RNA viruses, such as picornaviruses [55] and hepatitis C virus [51], while PAA is an inhibitor of DNA virus replication [50]. On the other hand, the RdRP of norovirus, which is also a (+)-RNA virus, has been reported to be sensitive to PAA but not gliotoxin [50]. The different sensitivity to RdRP inhibitors is possibly due to the *de novo* and polyadenylation-independent characteristic of nodaviral or noroviral RdRP [50]. Besides, the amino acid sequences of nodaviral RdRPs are partly similar to DNA polymerase by sequence analyses [41], which may be a cause for the inhibitor selectivity. Based on this and previous studies of FHV and WhNV, the *de novo* initiation may be a general mechanism in the family *Nodaviridae*.

The 3′-ends of RNA templates are of great importance for *de novo* initiation of RNA synthesis [28], [29], [30], [58]. For WhNV, the 3′-terminal 2 nucleotides of both (+)- and (−)-stranded RNA1 templates are required for synthesis initiation [41]. On the other hand, for FHV, the 3′-terminal 3 nucleotides of (−)-RNA1 template are required for synthesis initiation, while deleting 13 nucleotides from the 3′-end of (+)-RNA1 template only resulted in a reduction of RNA synthesis (Figure 6). This difference between WhNV and FHV might be due to the fact that the 3′-ends of (+)- and (−)-RNA1s of WhNV are similar in nucleotide sequence, while those of FHV are quite different. Moreover, the different requirement of FHV protein A to the 3′-ends of (+)- and (−)-RNA1 templates suggest that FHV protein A uses distinct strategies to initiate RNA synthesis on the (+)- and (−)-strand templates. Besides, previous study by Ball found that deletion of 5 nucleotides from 3′-end of FHV (+)-RNA1 transcript prevented RNA1 replication in cells [43], while our *in vitro* study found that deleting 13 or more nucleotides only resulted in a partial loss of RNA synthesis. This discrepancy is probably due to the different experimental conditions, as the former study used an FHV RNA1 replicon in baby hamster kidney 21 (BHK21) cells, while our *in vitro* experiment was conducted using purified recombinant FHV protein A and RNA1 template in the absence of other viral and cellular factors. Considering the importance of cellular factors such as intracellular membranes and host proteins on viral RNA replication [62], [63], the presence of cellular factors may further modulate FHV protein A for a better controlled RNA synthesis initiation.

The 3′-ends of viral RNAs may be subjected to degradation by cellular exonucleases [13], [58], [64], [65]. It has been previously reported that some viral RdRPs are also TNTases to add non-templated nucleotides to the 3′-end of viral RNA [33], [57], [58], [59], and the TNTase activity was often suggested as a rescue mechanism to repair the 3′-terminal loss of viral RNAs and restore viral RNA replication. However, since the RdRP and TNTase activities often share the same catalytic motif (GDD) in a single protein, it is hard to evaluate the role of one activity without involving the other. In this and the previous studies of WhNV protein A and FHV protein A, we designed a set of experiments to deliberately separate the TNTase and RdRP reactions, thereby successfully evaluating the potential terminal repair role of TNTase activity *in vitro*. Similarly with the RdRP activity, the 3′-end of RNA is also critical for the TNTase activity, as FHV protein A required the 3′-terminal 4 nucleotides for its TNTase activity (Figure 7A), which is slightly different with WhNV protein A whose TNTase requires 3′-terminal 2 nucleotides [41].

In this study, we characterized the RdRP activity of FHV protein A in detail and revealed that FHV protein A initiates RNA synthesis *de novo*, and also possesses TNTase activity. Although the exact role of TNTase is hard to be determined in cells due to technical limitations, we found that FHV protein A’s TNTase activity can repair the 3′ initiation site and rescue synthesis initiation at least *in vitro*. The current study, together with our previous study of WhNV protein A, suggests that the *de novo* initiation mechanism of RNA synthesis as well as the TNTase activity may generally apply to the family *Nodaviridae*, and represents an important progress in understanding the RNA replication of FHV, the well recognized model virus.
